# A Novel Quadrilateral Contour Disentangled Algorithm for Industrial Instrument Reading Detection

**DOI:** 10.3390/e27020122

**Published:** 2025-01-24

**Authors:** Xiang Li, Changchang Zeng, Yong Yao, Jide Qian, Haiding Zhang, Sen Zhang, Suixian Yang

**Affiliations:** 1 School of Mechanical Engineering, Sichuan University, Chengdu 610065, China; 2 School of Computer Science, Civil Aviation Flight University of China, Guanghan 618307, China; 3 National Institute of Measurement and Testing Technology, Chengdu 610056, China; 4 School of Big Data, Guizhou Institute of Technology, Guiyang 550003, China

**Keywords:** instrument reading detection, quadrilateral contour disentangled, MsFPN, PCDR, quadrilateral detector

## Abstract

Instrument reading detection in industrial scenarios poses significant challenges due to reading contour distortion caused by perspective transformation in the instrument images. However, existing methods fail to accurately read the display automatically due to incorrect labeling of the target box vertices, which arises from the vertex entanglement problem. To address these challenges, a novel Quadrilateral Contour Disentangled Detection Network (QCDNet) is proposed in this paper, which utilizes the quadrilateral disentanglement idea. First, a Multi-scale Feature Pyramid Network (MsFPN) is proposed for effective feature extraction to improve model accuracy. Second, we propose a Polar Coordinate Decoupling Representation (PCDR), which models each side of the instrument contour using polar coordinates. Additionally, a loss function for the polar coordinate parameters is designed to aid the PCDR in more effectively decoupling the instrument reading contour. Finally, the experimental results on the instrument dataset demonstrate that QCDNet outperforms existing quadrilateral detection algorithms, with improvements of 4.07%, 1.8%, and 2.89% in Precision, Recall, and F-measure, respectively. These results confirm the effectiveness of QCDNet for instrument reading detection tasks.

## 1. Introduction

Instrument reading detection is a crucial process to ensure the digital transformation of the manufacturing industry [1]. For instrument reading detection tasks in industrial scenarios, instrument images are captured using industrial cameras, inspection robots, handheld cameras, and surveillance systems. Challenges such as image blurring, distortion, and perspective transformations often arise due to varying imaging angles and illumination. These issues significantly impact the performance of existing detection algorithms, preventing the accurate identification of instrument reading regions. To address these challenges, this paper proposes a contour disentangled detection network based on computer vision techniques for instrument reading detection tasks.

**Figure 1:**
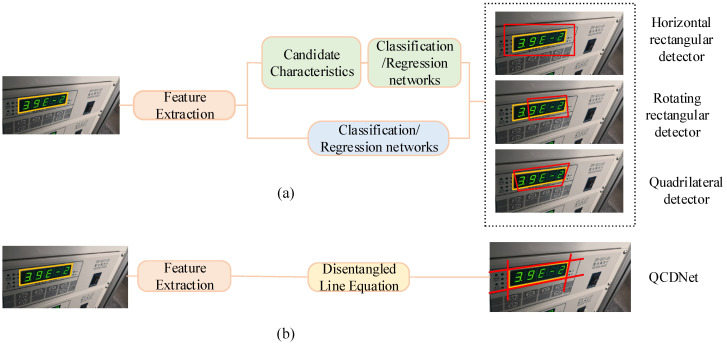
The representation method of instrument reading contour, where (**a**) is the existing three types of detectors and (**b**) is the contour disentangled detector.

With the widespread application of computer vision technology in image processing [2] and pattern recognition [3], object detection algorithms have gradually become effective tools in various domains, such as scene text detection [4], intelligent transportation [5], safety belt detection [6], and other fields. Instrument reading detection can be regarded as a generalized object detection problem. However, it differs from common object detection tasks due to unique challenges in distinguishing the foreground from the background. These challenges include interfering factors around the instrument display area, tilted instrument angles, and complex industrial environments. Specifically, the instrument images captured by industrial cameras are often affected by distortions resulting from rotational changes or perspective transformations. Such conditions pose significant challenges for instrument reading detection tasks. In this case, common object detection algorithms often struggle to accurately locate the reading contours, as illustrated in Figure 1. Existing detection algorithms tend to exhibit issues such as excessive inclusion of background information and insufficient capture of foreground details.

Common object detection algorithms are typically categorized into horizontal rectangular detectors, rotating rectangular detectors, and quadrilateral detectors. Among these, the “R-CNN family” [7,8,9] represents the most prominent two-stage approach for horizontal rectangular detection. This framework employs thousands of class-independent anchor boxes to facilitate object detection. While two-stage methods generally offer higher accuracy, they come at the cost of increased computational complexity [10]. Representative one-stage horizontal rectangular detectors include YOLO [11], SSD [12], and RetinaNet [13]. These methods operate by directly dividing the input image into small grids, predicting the bounding boxes for each grid, and subsequently refining these predictions to match the ground-truth boxes. In recent years, horizontal rectangular detectors have been widely used in instrument reading detection [14,15], scene text detection [16], remote sensing images [17], and other related scenarios. However, these detectors generate bounding boxes aligned along the horizontal axis, which restricts their effectiveness in many real-world applications. In practical applications, target objects are often densely arranged, with a large aspect ratio, and undergo rotational transformations. Therefore, the rotating rectangle detector emerged to solve the problem of rotation transformation. The rotating rectangular detector adds angle information on the basis of the horizontal rectangular detector, which is also relatively common in remote sensing image [18,19,20], scene text detection [21,22], and other tasks. The predicted substances include category, position coordinates, length, width, and angle, which make it more accurate than the horizontal rectangular detector. However, in practical applications, target objects often experience varying degrees of perspective transformation due to camera angle variations, which poses additional challenges. Horizontal and rotating rectangular detectors cannot accurately locate the bounding box of the target objects. In this case, the quadrilateral detector can more accurately detect the boundary box of the target objects. Consequently, some quadrilateral detection algorithms have been proposed [23,24,25]. The difficulty of the quadrilateral detection algorithm mainly comes from its four twisted sides, which are irregularly and independently arranged. Quadrilateral detection algorithms typically predict bounding boxes by regressing the coordinates of four vertices, with each vertex simultaneously influencing the positioning of two adjacent edges. As a result, the sides of the quadrilateral contour are affected by two vertices, and the adjacent side is also affected to varying degrees, which is a vertex entanglement problem. This problem suppresses the learning efficiency and detection performance of the quadrilateral detector. For the entangled vertices problem, researchers have proposed solutions [26,27,28,29] which alleviate such problems by setting an appropriate vertex sequential protocol. However, these approaches primarily serve as remedial measures rather than fundamentally solving the problem.

**Figure 2:**
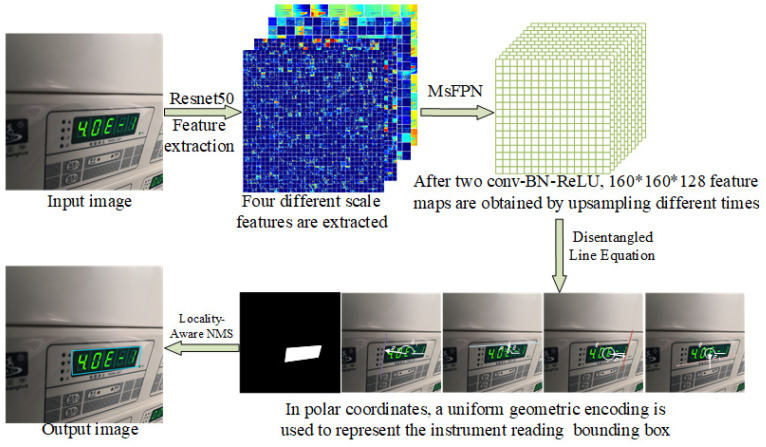
Flowchart of the QCDnet method.

To address the challenges of distorted reading contours and vertex entanglement in instrument images, this paper proposes an instrument reading detection network based on quadrilateral contour disentanglement (QCDNet). In QCDNet, the residual network is employed as a fundamental module for feature extraction from instrument images. Then, a Multi-scale Feature Pyramid Network (MsFPN) module is developed to integrate low-level and high-level features for strong semantic feature information. Meanwhile, Polar Coordinates Decoupling Representation (PCDR) is introduced, which decouples each side of the instrument reading contour from the overall structure, as shown in Figure 2. Additionally, the Polar-IoU and cosine angle loss function are designed to optimize the characterization parameters of each side’s geometric properties, enabling better representation in instrument images. Therefore, QCDNet can effectively solve the problem of distorted reading contours and vertex entanglement, offering a reliable solution for instrument reading detection in industrial scenarios. This paper provides a reference model for enhancing the efficiency and accuracy of instrument readings detection in industrial scenarios.

In summary, the main contributions of this work are as follows:

(1) A residual network combined with an MsFPN module is proposed to fuse low-level features and high-level features to obtain strong semantic feature information. The ablation experiment results prove the validity of MsFPN in the Instrument Dataset.

(2) A novel Polar Coordinate Decoupling Representation method is introduced, which disentangles each side of the instrument reading contour using polar coordinates. Based on the geometrical properties of instrument reading contour, the Polar-IoU and cosine angle loss functions are designed to enhance the model learning capability and decoupling performance.

(3) Extensive validation experiments were conducted on the Instrument Dataset. The experimental results demonstrate that the proposed QCDNet outperforms comparative methods in the instrument reading detection task.

## 2. Related Work

Based on the above introduction, it can be inferred that addressing the distortion of the reading area contour caused by rotation and perspective transformation is a crucial challenge in instrument reading detection tasks. One of the solutions to this issue is the quadrilateral detector. Recent studies [26,28,30] have highlighted that the quadrilateral bounding boxes serve as a key representation for multi-directional detection algorithms. However, a significant challenge in the generation process of quadrilateral bounding boxes is the vertex entanglement problem. Existing quadrilateral detectors can be broadly categorized into anchor-free and anchor-based methods, depending on whether anchors are utilized. For the anchor-free quadrilateral detectors [31,32,33], the approaches involve detecting the corner points of the target and subsequently generating bounding boxes based on these points. While this method avoids reliance on predefined anchors, it often involves complex post-processing and is highly susceptible to outliers because anchor-based quadrilateral detectors [21,26,34,35] directly learn the bounding boxes to locate the target object, reducing the complexity of post-processing. However, these detectors are sensitive to label sequences, which can affect detection accuracy.

Most of the above quadrilateral detectors directly or indirectly rely on learning four points to locate the bounding box of the target object. However, the quadrilateral bounding box is determined with four points, and their order can easily become inconsistent during training. Moreover, the four vertices of the quadrilateral bounding box are highly sensitive to the label sequence. It is difficult for the network to determine the order of the four vertices, and a small disturbance changes the whole sequence completely. Therefore, it is crucial to establish a sequential protocol prior to training. Existing solutions for mitigating label inconsistency are summarized as follows.

**Figure 3:**
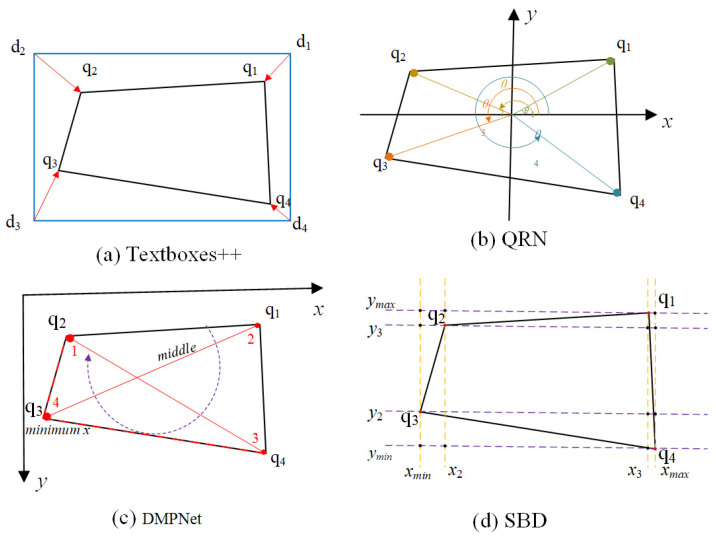
Previous solutions are negatively affected by the label inconsistency problem.

Textboxes++ [26] solved the label inconsistency problem by implicitly regressing the four vertices of the target quadrilateral, as shown in Figure 3a. Specifically, the algorithm determines the sequence by calculating the distance between each quadrilateral vertex q*_i_* and the corresponding horizontal rectangular vertex d*_i_* (*i* = 1, 2, 3, 4).

QRN [27] resolved the vertex ordering issue by sorting the vertices of the target quadrilateral and reference rectangle in ascending polar order, as shown in Figure 3b. Specifically, QRN computes the average center point of the four vertices and constructs a Cartesian coordinate system. It then calculates the intersection angles of the four vertices with the origin and selects the vertex with the smallest angle as the starting point.

DMPNet [28] introduced a slope-based method for determining label sequences, as shown in Figure 3c. The first point is determined by the minimum value of x in the four vertices, and then the minimum point is connected to the other three points. The point on the other end of the middle line is the third point, the point on the top left of the middle line is the second point, and the other point is the fourth point. Finally, the slope between the two diagonal lines, 13 and 24, are compared. The vertex with the smaller *x*-coordinate on the line with the larger slope is selected as the new first point. The above procedure is then repeated to identify the remaining three points in sequence.

SBD [29] proposed a matching learning module to solve the label inconsistency problem, as shown in Figure 3d. The method begins by plotting the *x*-axes and *y*-axes for each vertex of the quadrilateral. These axes are then sorted in ascending order, and their intersection points with the bounding box are marked. Next, the first point (shown as a red dot q3) is identified at the intersection of the bounding box and the axis with the minimum *x*_min_ value. Finally, the remaining three points are labeled sequentially in a clockwise direction.

Although the above sequential protocols can alleviate label confusion to a certain extent, their performance deteriorates significantly when the instrument image is severely distorted by perspective transformations. Moreover, these methods often involve complex computations. In contrast, the proposed QCDNet focuses on decoupling each side of the instrument reading contour and representing the line equations of each side using polar coordinates. This approach minimizes the reliance on complex label sequential protocols. Additionally, it eliminates the need for intricate post-processing steps to generate quadrilateral bounding boxes, effectively resolving the vertex entanglement problem.

## 3. Proposed Method

**Figure 4:**
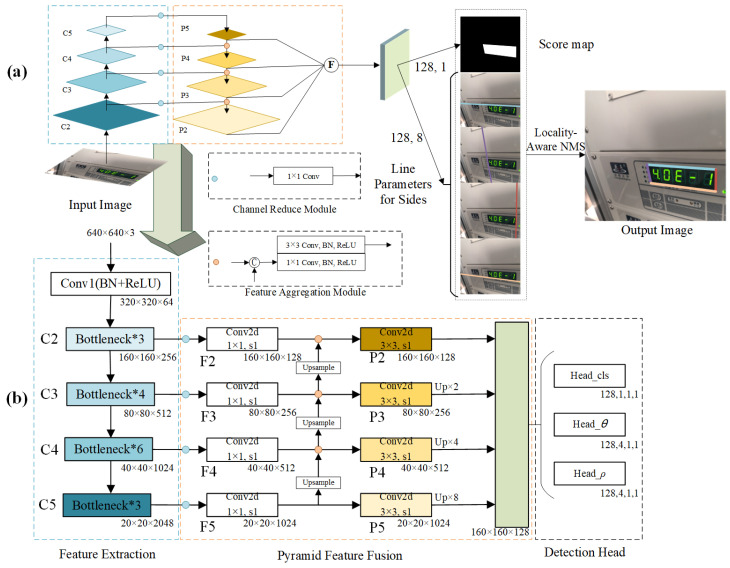
The architecture of QCDNet in instrument reading detection.

The proposed QCDNet is designed for instrument reading detection based on quadrilateral contour disentanglement, and its structure, is shown in Figure 4a. First, inspired by Feature Pyramid Network (FPN) [36], a Multi-scale Feature Pyramid Network based on residual connection network is introduced to extract strong semantic feature information. Second, a polar coordinate decoupled representation is proposed to represent the line equations of each side of the instrument reading contour in polar coordinates, which can effectively solve the quadrilateral vertex entanglement problem. Then, a suitable loss function is designed for the corresponding polar coordinate parameters to better decouple each side in the contour. Finally, the corresponding label generation method is provided. The detailed implementation of these components is detailed in the following subsections.

### 3.1. Multi-Scale Feature Pyramid Network

QCDNet adopts a fully convolutional structure for quadrilateral bounding box regression. Its architecture is based on encoder–decoder design, utilizing the pre-trained ResNet50 backbone. The kernel configuration of the architecture is illustrated in Figure 4b. To enhance feature effectiveness, feature maps C2, C3, C4, and C5 are extracted from the backbone network. For 640 × 640 input images, the corresponding output feature map sizes are 160 × 160 × 256, 80 × 80 × 512, 40 × 40 × 1024, and 20 × 20 × 2048, respectively. These multi-scale feature maps are subsequently processed through the MsFPN after down-sampling, enabling the network to effectively learn features at different scales.

The F*_i_* values represent the features under different receptive fields. The normal feature F*_i_*_−1_ and the up-sample feature F*_i_* (*i* = 3, 4, 5) values are generated using bilinear up-sampling. Subsequently, F*_i_*_−1_ is fed into the feature aggregation module for further processing. After applying two Conv-BN-ReLU function modules to F*_i_*, the intermediate features, P*_i_*_−1_, can be obtained. To achieve high-level semantic representation extraction, the four feature maps (P2, P3, P4, P5) are fused by up-sampling the function F for different times. The formula can be described as follows.

(1) $$F=P_{2}∥Up_{\times 2}(P_{3})∥Up_{\times 4}(P4)∥Up_{\times 8}(P5),$$

where || is the concatenation function, and Up represents the up-sample operation where the indicator “×2”, “×4”, “×8” corresponds to the operand of 2, 4, and 8, respectively.

Finally, the output channel information includes a text mask that focuses on the meter reading area and a series of polar parameters. These outputs are visualized in Figure 4a using different colors to represent various components.

### 3.2. Polar Coordinates Decoupling Representation

In industrial scenarios, instrument images are often distorted due to the imaging angle, a phenomenon referred to as affine transformation. The image distortion causes the instrument reading contour to appear in an arbitrary quadrilateral shape. The degree of deformation of an arbitrary quadrilateral has intrinsic geometric constraints that are observable in the Cartesian coordinate system. It can be intuitively observed that each side is affected by two vertices, which is the vertex entangled problem mentioned in the introduction. Inspired by previous studies [25,37,38], this paper attempts to represent the instrument reading quadrilateral bounding box in polar coordinates with a unified geometric encoding. This approach effectively avoids the vertex entanglement problem. Each side of the quadrilateral bounding box, being an independent straight-line equation, can be represented by a consistent formula as follows.

(2) Ax+By=−C,

where *A*, *B*, and *C* are constants, and *C* cannot be 0. When *C* = 0, there are multiple solutions to the linear equation. This results in *A*, *B*, and *C* not having physical meaning in the instrumentation image. It is difficult for the network to learn the correlation between the data, which is not conducive to model training.

**Figure 5:**
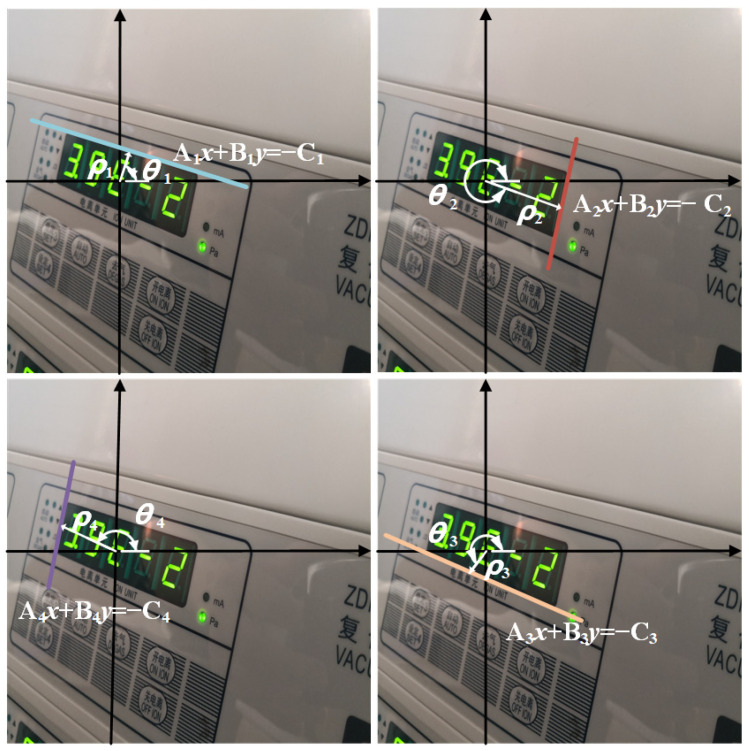
Representation of linear equation.

In response to the above, the linear equation needs to be transformed into the polar coordinate equation, as formulated in Equations (3) and (4). Additionally, a representation protocol is defined, where the four sides of the instrument reading bounding box are outlined in a clockwise direction, starting from the top-left corner and following the order of the annotated vertices, as shown in Figure 5.

(3) $$\left\{\begin{array}{l} x=\rho \cos\theta \\ y=\rho \sin\theta \end{array}\right.,$$

(4) xcosθ+ysinθ=ρ,

In Figure 5, *ρ* and *θ* are two independent parameters. The *ρ* is the closest distance from the origin to the line equation, and *θ* is the rotation angle from the positive direction of the *x*-axis to the closest distance. From the figure, it can be seen that a line needs three parameters, A, B, and C, to be represented in Cartesian coordinate system. In contrast, only *ρ* and *θ* are needed in polar coordinate system. Therefore, the proposed method reduces the number of parameters. The most important thing is that *ρ* and *θ* have geometric properties in the image, which is beneficial for network learning.

In the inference stage, the initial *A*, *B*, and *C* values of the Cartesian coordinate line equation at the corresponding position are first calculated by the polar coordinate equation, and the calculation formulas are expressed as follows.

(5) $$\left\{\begin{array}{l} A=\cos\theta \\ B=\sin\theta \\ C=-\rho \end{array}\right.,$$

Secondly, taking the lower-left corner of the instrument image as the global origin, the linear parameters in Equation (5) are transformed to align with the global origin. Subsequently, the four lines intersect pairwise in Cartesian coordinate system to obtain four intersection points, (*x_i_*, *y_i_*) *i* = 1, 2, 3, 4. We calculate one of the intersections using the linear equations A_1_*x* + B_1_*y* = −C_1_ and A_2_*x* + B_2_*y* = −C_2_ as an example. The calculation formulas are Equations (6)–(8). It is worth noting that the parameter *D* cannot be equal to 0 to ensure the validity of the equation.

(6) $$D=A_{1}B_{2}-A_{2}B_{1},$$

(7) $$x_{i}=\frac{B_{1}C_{2}-B_{2}C_{1}}{D},$$

(8) $$y_{i}=\frac{A_{2}C_{1}-A_{1}C_{2}}{D},$$

In summary, to facilitate model training, the linear equation of the instrument boundary box is transformed into the corresponding polar coordinates. During inference, the *ρ* and *θ* parameters are first converted to the line parameters of global Cartesian coordinates. Then, the bounding box of the instrument reading can be formed after sequentially calculating the intersections between the lines. Finally, the detection box is obtained through a simple Locality-Aware NMS operation.

### 3.3. Loss Functions

The key for QCDNet is to accurately detect the contours of instrument reading depending on the expression of the line equations with ρ and θ, which avoids the vertices entanglement problem. Therefore, it is particularly important to design an appropriate loss function. The overall loss function of the model can be expressed as follows.

(9) $$L=\lambda_{cls}L_{cls}+\lambda_{\rho }L_{\rho }+\lambda_{\theta }L_{\theta },$$

where *L_cls_* represents the reading/non-reading region classification loss. *L_ρ_* and *L_θ_* denote the regression loss of line parameters ρ and θ in polar coordinates, respectively. *λ_cls_*, *λ_ρ_*, and *λ_θ_* are used to balance the weight of those losses. In this paper, we set the three parameters to 1.

To overcome the challenge of instrument reading detection caused by affine and perspective transformation, Polar-IoU loss, motivated by IoU loss [39], is proposed in L*ρ*. The formulation can be described as follows.

(10) $$L_{\rho }=-\frac{1}{N}\sum_{i}\log\frac{\max({\hat{\rho }}_{i},\rho_{i})-\min({\hat{\rho }}_{i},\rho_{i})}{\max({\hat{\rho }}_{i},\rho_{i})},$$

(11) $$\rho_{i}=\frac{\left|C\right|}{\sqrt{A^{2}+B^{2}}},$$

where N is the number of pixels in the valid reading area. $\rho_{i}$ and ${\hat{\rho }}_{i}$ represent the label and prediction of *ρ* in the *i*th N location, respectively. The polar diameter $\rho_{i}$ can be calculated using the shortest-distance formula. The formula can be expressed as (11). Compared with L_1_, L_2_, and Smooth L_1_ loss [8], the proposed Polar-IoU loss normalizes polar diameters across various directions. This normalization enables the model to better handle multi-scale detection, thereby enhancing its robustness and accuracy.

Considering the cyclic nature of the polar angle *θ*, which is in the range of [0, 2π] in the polar coordinate system, the loss decreases as the polar angle difference increases from 0π to 2π. The proposed cosine angle loss *L_θ_* is inspired by EAST [21]. The constructed loss function is shown in Equation (11), where $\theta_{i}$ and ${\hat{\theta }}_{i}$ denote the label and the predicted *θ* at the *i*th angle, respectively.

(12) $$L_{\theta }=1-\frac{1}{N}\sum_{i}\cos\frac{\left|{\hat{\theta }}_{i}-\theta_{i}\right|}{2},$$

The label angle *θ* is defined based on different locations within the instrument reading area. Suppose there is any point (*x*_0_, *y*_0_) in the instrument reading area. We use the four vertices (*x_i_*, *y_i_*) *i* = 1, 2, 3, 4 of the instrument readings contour. The parameters A, B, and C of the linear equation can be calculated. Meanwhile, the angle label $\theta_{i}$ can be formed by calculating the angle between the unit vector $\vec{e}$ in the positive direction of the *x*-axis and the vertical vector $\vec{l}$, as formulated in Equation (13). According to these equations, it is worth noting that parameter BC is greater than 0, and $\theta_{i}$ is under the *x*-axis. In this condition, this needs to be subtracted from 2π to obtain the true label $\theta_{i}$.

(13) $$\left\{\begin{array}{l} \vec{l}=(\frac{-AC}{A^{2}+B^{2}},\frac{-BC}{A^{2}+B^{2}})\\ \vec{e}=(1,0)\\ \theta^{i}=\arccos\frac{\vec{l}\cdot \vec{e}}{\left|\vec{l}\right|\left|\vec{e}\right|} \end{array}\right.,$$

The classification task in this paper is framed as a binary classification problem, distinguishing between foreground and background regions. Specifically, the instrument reading area is designated as the foreground, while all other areas are classified as the background. Therefore, to enhance the model’s classification capability, the loss function *L_cls_* employs Binary Cross Entropy Loss (BCE) and Online Hard Example Mining (OHEM) [40]. The OHEM mechanism selects the most challenging samples during training, effectively improving model robustness. The loss function is defined in Equation (14), where $y_{i}$ and ${\hat{y}}_{i}$ denote the label and the predicted value at the *i*th position, respectively. Note that N is actually the number of valid text pixels.

(14) $$L_{cls}=-\frac{1}{\left|N\right|}\sum_{i\in N}\left[y_{i}\log{\hat{y}}_{i}+(1-y_{i})\log(1-{\hat{y}}_{i})\right],$$

**Figure 6:**
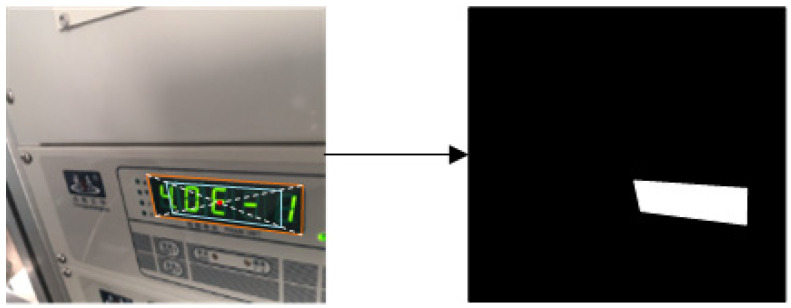
Generation of instrument reading mask.

For classification label generation, considering that the *ρ* of the instrument reading edge area is difficult to distinguish, we scale down the mask of the instrument reading area proportionally along the diagonal. Here, the scaling factor is 0.35 [25]. As shown in Figure 6, the model produces segmentation results, where the ground truth for the instrument reading area is represented in binary form. In this representation, foreground pixels corresponding to the instrument reading area are labeled “1”, while background pixels are labeled “0”.

## 4. Experiments

In this section, we first describe the preparation and implementation details of the experiments. Then, the performance of functional modules is analyzed through ablation experiments. Finally, the effectiveness of the proposed model is compared with mainstream methods on an instrument reading detection task through quantitative and qualitative experiments.

### 4.1. Dataset Preparation

**Figure 7:**
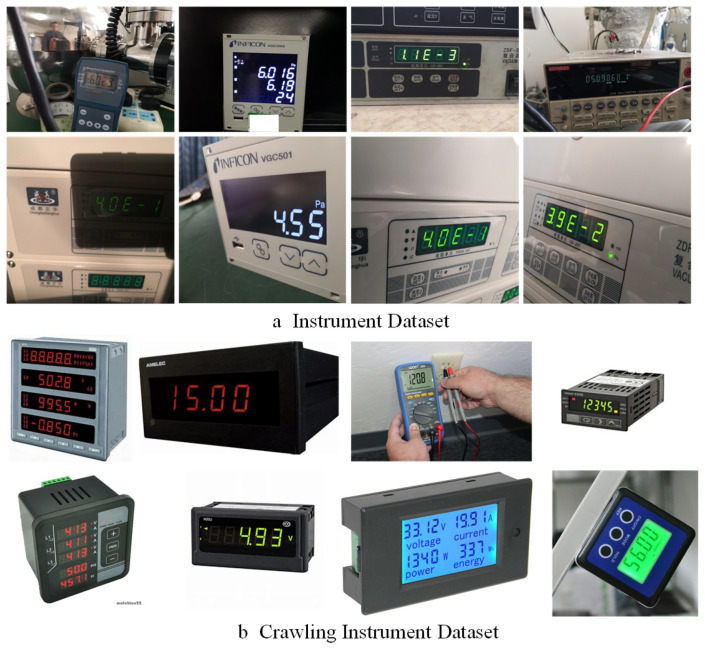
(**a**) is the instrument dataset of different challenging environments in real industrial scenarios, and (**b**) is the instrument dataset found on the Web.

Considering the fact that no publicly available instrument image datasets have been reported so far, 1723 instrument images with a variety of lighting conditions, backgrounds, imaging angles, and resolutions were collected in industrial scenes for this study. These images were scaled to 1000 × 1000 pixels using a bilinear interpolation method to obtain the Instrument Dataset for experiment analysis, as shown in Figure 7a. Meanwhile, in order to improve the training efficiency of the deep learning model, 10,520 instrument images were collected from the Internet, a collection called the Crawling Instrument Dataset, as shown in Figure 7b. Both datasets were manually labeled using two different tools (LabelImg and VIA). The difference between the two annotation tools is that LabelImg uses a center point, height, and width annotation method, and the bounding box is a rectangle. VIA uses the annotation method of four vertices, and the bounding box is an arbitrary quadrilateral box. According to experimental needs, VIA is most used as the labeling tool for quadrilateral detectors, while LabelImg is often used for horizontal and rotation detectors. In our datasets, two annotation tools were used for the Instrument Dataset, and the Crawling Instrument Dataset was only annotated using the VIA. In the experiment, the labeled Crawling Instrument data were used to pre-train the model, while the Instrument Dataset was employed for the model training and testing with 8:2 ratios.

### 4.2. Implementation DETAILS

The Adam [41] optimization algorithm was employed for model training, with an initial learning rate set to 1 × 10^−3^, dynamically adjusted based on the number of iterations. Specifically, the model pre-training was conducted on the Crawling Instrument Dataset, where the initial learning rate decay was 1 × 10^−4^ at 40,000 iterations. Subsequently, the model was fine-tuned and optimized on the Instrument Dataset over 60,000 iterations with a constant learning rate. After completing the final epoch, the model parameters were fixed to evaluate its performance on the test dataset. To ensure fairness in the experiment, all models were run in the same environment: Intel Xeon(R) W 2145@3.7 GHz CPU, NVIDIA Quadro RTX4000 GPU, Ubuntu 20.04, CUDA 11.2, and Pytorch 1.7.

During the training phase, the height and width of the instrument images were randomly scaled in the range of [640, 2560]. This scaling was implemented to account for the varying distorted shapes of the instrument reading areas. Additionally, the brightness, contrast, saturation, and color channels of the instrument image were randomly changed to improve the generalization ability of the model. Finally, 640 × 640 patches were randomly cropped from the transformed instrument images to serve as the training data.

### 4.3. Evaluation Protocols

In order to evaluate the detection performance of the algorithm on the instrument image dataset, we used Average Precision (AP), Precision, Recall, F-measure, and inference time (ms) as evaluation protocols. The formulas are as follows.

(15) $$\mathrm{AP}=\int_{0}^{1}P(R)d(R),$$

(16) $$Precision=\frac{\mathrm{TP}}{\mathrm{TP}+\mathrm{FP}},$$

(17) $$Recall=\frac{\mathrm{TP}}{\mathrm{TP}+\mathrm{FN}},$$

(18) $$\text{F-measure}=\frac{2Precision*Recall}{Precision+Recall}$$

where TP, FN, and FP are true positives, false negatives, and false positives, respectively. In the detection task, a detected bounding box is considered true if its Intersection over Union (IoU) with the ground-truth bounding box exceeds a threshold. Incorrect bounding box predictions are counted as false positives, while false negatives are the bounding boxes that should have been detected but were missed. F-measure is used to measure the overall performance of the model.

### 4.4. Validation of Model Effectiveness

In order to verify the effectiveness of our proposed method in instrument reading detection tasks, some experiments were implemented for performance analysis regarding the following three aspects: (1) backbone network, a strong feature extraction network that can significantly enhance the performance of the algorithm; (2) ablation experiments, in which the effects of the proposed MsFPN, Polar-IoU loss, and cosine angle loss are analyzed by the control variable method; and (3) qualitative and quantitative analysis for the detector, in which the effectiveness of QCDNet in instrument reading detection is demonstrated through both qualitative and quantitative evaluations.

#### 4.4.1. Backbone Network

**Figure 8:**
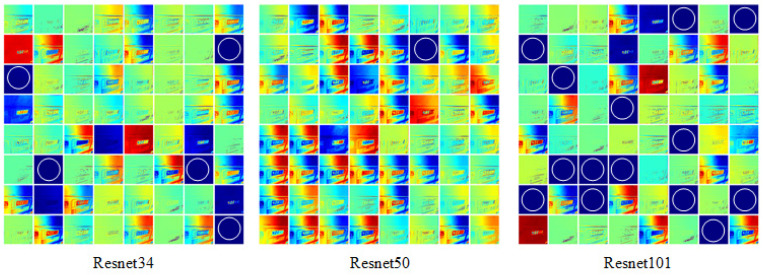
Visualization of the feature maps of the f2_BN1 layer of the three backbone networks. The white circle indicates the feature map that has not been clearly extracted from the instrument readings.

In computer vision tasks, the network responsible for image feature extraction is called the backbone network. Commonly used CNN backbone networks include VGG [42], GoogLeNet [43], and ResNet [44], etc. Traditional CNN convolutional layers or fully connected layers often face issues such as information loss and high computational cost during information transmission. ResNet has the ability to solve problems such as gradient disappearance and network degradation, so we employ this structure as the backbone network in our network design. A deeper network typically offers better performance than a shallower one. However, merely increasing the number of layers can lead to higher computational costs without guaranteeing improved performance. To determine the optimal backbone network for our model, we compared the feature extraction performance of ResNet34, ResNet50, and ResNet101 by visualizing the feature maps. Specifically, we selected the feature map of the f2_BN1 layer 8 × 8, shown in Figure 8. The rationale for choosing this layer is that its advanced features capture macro-level information of the instrument image, which might not be easily recognizable by humans. The f2_BN1 layer represents finer details of the instrument image. By observing the feature maps, we can evaluate the performance of the backbone network in extracting instrument reading features. From Figure 8, it can be seen that only one feature map of the ResNet50 network does not clearly extract instrument image features, while ResNet34 and ResNet101 have 5 and 14 feature maps that do not extract instrument image features, respectively. So ResNet50 was selected as the backbone network for our model.

#### 4.4.2. Ablation Experiment

To verify the effectiveness of the proposed QCDNet detection network, ablation experiments were conducted. Specifically, QCDNet integrates three designed modules: MsFPN, Polar-IoU loss, and cosine angle loss. The impact of each module was analyzed using the control variable method to isolate their effects. The baseline model was derived from QCDNet by systematically replacing or removing specific modules. For instance, when evaluating the performance of Polar-IoU loss, the baseline model employed the commonly used L1 loss instead. Similarly, the effects of the other modules were analyzed by modifying the baseline configuration accordingly. The F-measure was utilized as the evaluation metric to measure the performance of the model in each scenario.

The results of the ablation experiment on the instrument dataset test set are shown in Table 1. It can be seen that the MsFPN module was added to integrate multi-scale features, which increased by 3.04% compared with the F-measure of the baseline model, indicating that the designed MsFPN has good competitiveness. Compared with the feature fusion method, the design of the boundary box loss function is also particularly important. Polar-IoU loss and cosine angle loss were designed for this paper, which independently predict each edge to avoid vertex entanglement. It can be seen from Table 1 that Polar-IoU loss and cosine angle loss significantly improved the detection performance of the model. The F-measure value increased by 5.44% and 4.74%, respectively, compared with the baseline model. Finally, the overall performance of the QCDNet model was further improved to 94.89% with the combined effect of the three modules, which is 7.48% better than the baseline model. The above ablation experiments verify the effectiveness of the proposed MsFPN, Polar-IoU loss, and cosine angle loss for bounding box detection.

**Figure 9:**
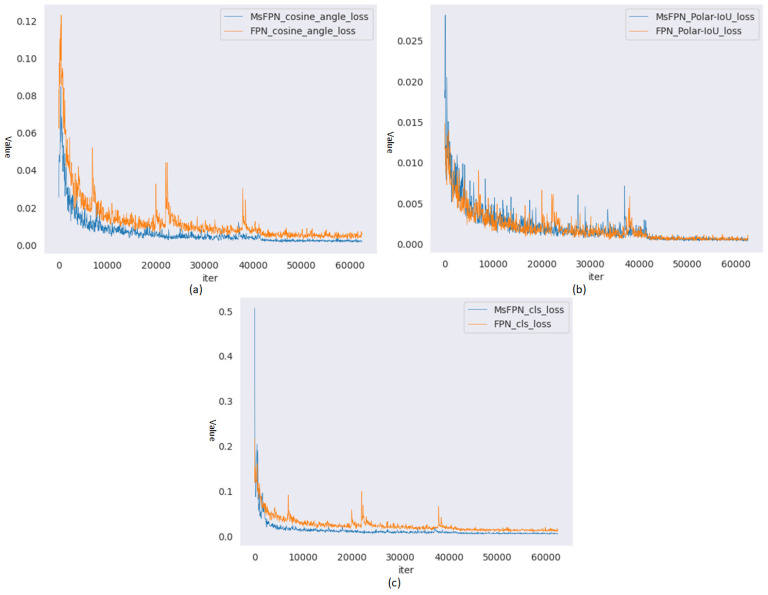
Iteration curves of loss function: (**a**) cosine angle loss; (**b**) Polar-IoU loss; (**c**) cls loss.

To validate the improvement in network performance obtained through MsFPN, we conducted ablation experiments on the Instrument Dataset test set. The experiments used Resnet50 as the feature extraction network to compare with the original FPN. Experimental results are shown in Table 2. When IoU was 0.50, the AP values of both MsFPN and FPN reached 1. However, when IoU was 0.85, the proposed MsFPN was 1.19% higher than the FPN. Furthermore, we visualized three loss curves for both methods during training, as presented in Figure 9. From the figure, it can be observed that the three loss curves of MsFPN are smoother and more stable than those of FPN. Therefore, the above ablation experiments validate the effectiveness of MsFPN. This also verifies the feasibility of the designed cosine angle loss and Polar-IoU loss in combination with other networks.

#### 4.4.3. Qualitative and Quantitative Results

To perform a comprehensive analysis and evaluation of the proposed method, qualitative and quantitative analyses were conducted using three different types of detectors. These analyses aim to verify the effectiveness of the proposed approach in accurately detecting instrument readings.

1. QCDNet vs. Horizontal Rectangle Detector:

For the horizontal rectangle detector, we choose an SSD and YOLO algorithm with higher accuracy and faster speed, RetinaNet, which solves the problem of data imbalance, and Faster-RCNN as the comparison method. To ensure fairness, we picked the instrument images with no affine transformation for model testing. As a rule of thumb, when the IoU was 0.5, the AP50 values of the five methods were equal to 1, which showed good performance, as shown in Table 3. For a more detailed comparison, we increased the IoU to 0.85. At this threshold, the RetinaNet algorithm achieved the best performance, followed closely by our proposed method, with the AP85 values differing by only 0.0037. The IoU threshold for standard target detection is typically set to 0.5, but higher thresholds require more robust and efficient detectors. To further evaluate performance, we calculated the AP values across a range of IoU thresholds [0.5, 0.95] at intervals of 0.05, averaging these 10 AP values for the final result. This method is derived from the evaluation method of the VOC dataset. Under this more rigorous evaluation method, the proposed method had the highest AP value, which was 0.42% higher than the SSD algorithm, as shown in Table 3. These results demonstrate the effectiveness of the proposed method for instrument reading detection tasks.

2. QCDNet vs. Rotated Rectangle Detector:

Rotating rectangles are special example of quadrilaterals which are more flexible than horizontal rectangles and represent instrument reading bounding boxes in any direction. We selected five state-of-the-art rotation detectors available at the time for comparison. Compared to horizontal rectangle detectors, distinguishing the performance of rotation detectors at an IoU of 0.5 is more challenging. Therefore, we calculated Recall, Precision, and F-measure for IoU thresholds of 0.5 and 0.85, respectively. The comparison results are summarized in Table 4. At IoU = 0.5, the proposed method achieved the highest Precision value of 0.9983, while its Recall and F-measure were slightly below those of Oriented R-CNN. However, at IoU = 0.85, the proposed method outperformed all five rotated rectangle detection methods in Recall, Precision, and F-measure. At this higher IoU threshold, the performance gap between quadrilateral detectors and rotated rectangle detectors became more pronounced. This confirms that the detector with the quadrilateral representation is more accurate than the rotated rectangle detector.

**Figure 10:**
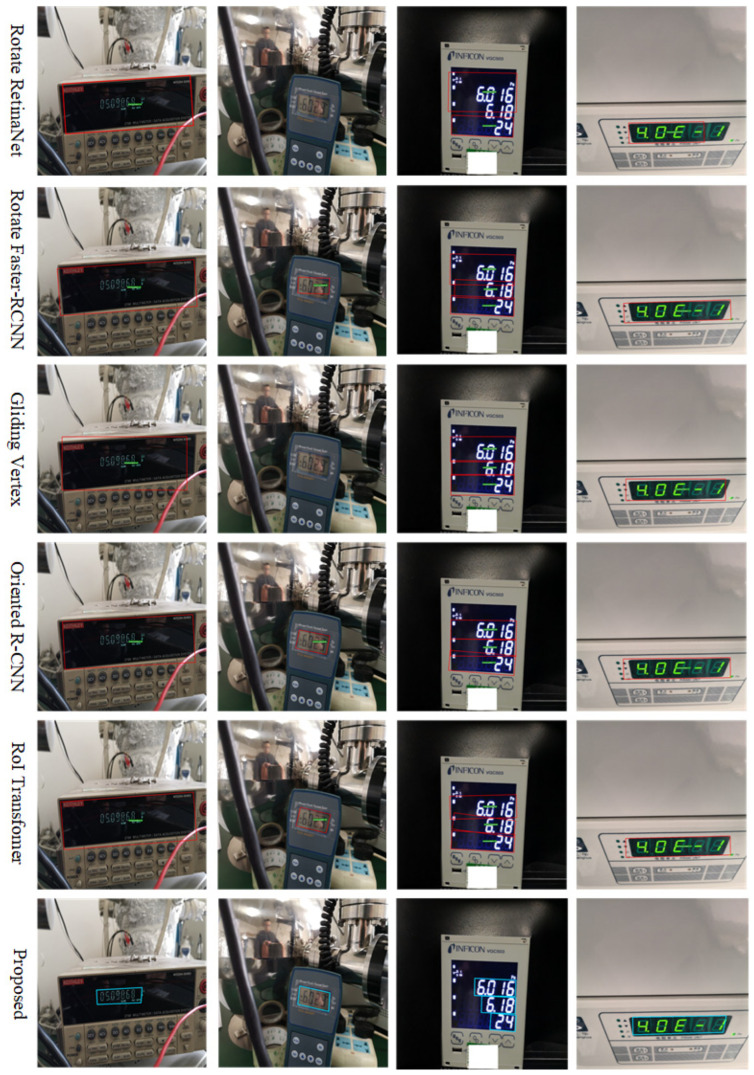
Detection results of the rotating rectangle detector and the proposed method on the Instrument Dataset test set.

Finally, the instrument reading detection results of six methods were visualized on the instrument images, as shown in Figure 10. From the figure, it can be seen that the five types of rotating rectangular detectors contain a lot of background information inside and the detection box is too large. The Rotated RetinaNet and Gliding Vertex algorithms failed to detect the reading in the second instrument image. For multiple-row instrument readings, as shown in the third column instrument image of Figure 10, the detection boxes of Rotated Faster-RCNN, Oriented R-CNN, and RoI Transformer overlapped and interleaved, making it impossible to accurately detect the instrument reading bounding boxes. However, the proposed method can accurately detect the bounding box of the instrument reading, even in the presence of rotation or affine transformations in the instrument images. Therefore, the above experiments verify that the proposed method can solve the problem of instrument image distortion caused by rotation and affine transformation.

**Figure 11:**
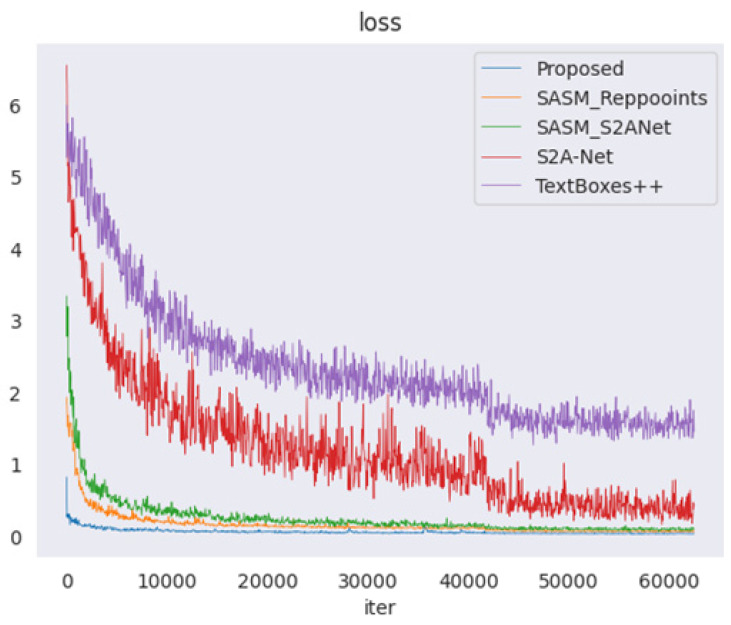
Iteration curves of loss functions of five quadrilateral detectors.

3. QCDNet vs. Quadrilateral Detector:

In real industrial scenarios, perspective transformation caused by the shooting angle often results in the distortion of instrument images. In this case, horizontal rectangle detectors or rotating rectangle detectors cannot accurately detect the instrument reading area. Therefore, the study of quadrilateral detectors is imperative. Existing quadrilateral detectors can be classified into anchor-based and anchor-free object detection algorithms according to whether there is a prior box. Most anchor-based quadrilateral detectors have the problem of vertex entanglement, and current solutions employ complex sequential protocols to alleviate this problem. The anchor-free quadrilateral detector eliminates the anchor generation mechanism and speeds up the model training, and these methods have higher accuracy. In this comparison test, we selected four quadrilateral detectors that were optimal at that time. Firstly, we compared the loss curves of the five detectors as shown in Figure 11. The loss values of TextBoxes++ and S2A-Net exhibited significant jitter and slow convergence rates. However, the proposed method demonstrated the fastest convergence speed, stabilizing after approximately 10,000 iterations. This indirectly validates the effectiveness of the proposed Polar-IoU loss and cosine angle loss for instrument reading detection. Subsequently, a quantitative comparison between the proposed method and other four quadrilateral detectors was performed, as shown in Table 5. The Recall and Precision values of the proposed method surpassed those of the second-best detector, SASM_Reppoints, by 1.8% and 3.07%, respectively. Additionally, the F-measure of the proposed method exceeded that of SASM_Reppoints by 2.89%. It is worth noting that the three evaluation indicators of the proposed method are all more than 10% higher than the TextBoxes++ algorithm.

**Figure 12:**
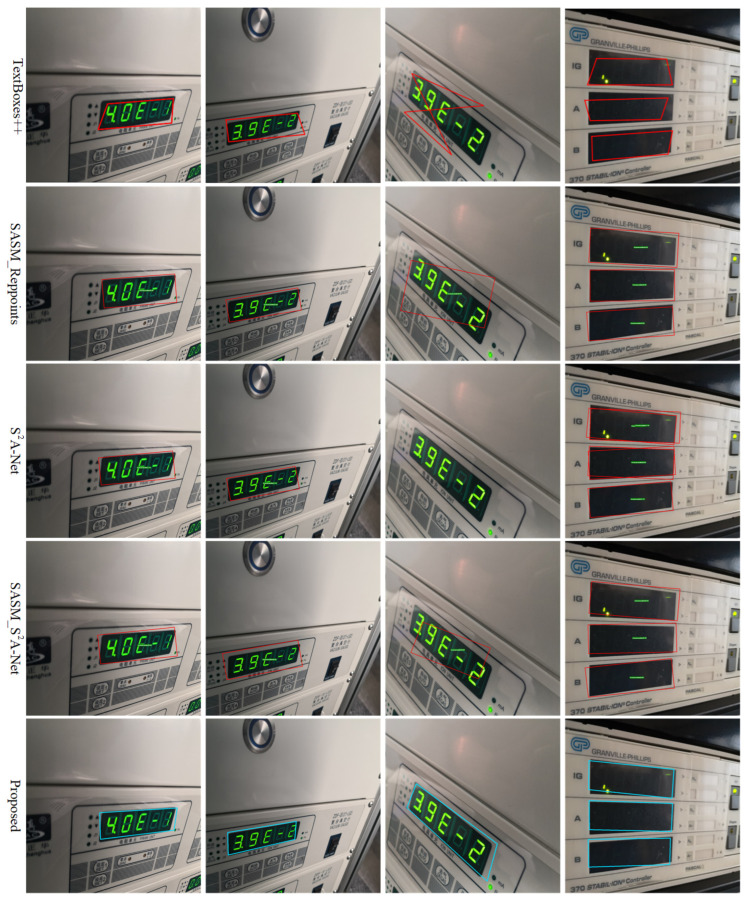
Detection results of five quadrilateral detector methods on the Instrument Dataset test set.

Moreover, from Table 5, it can be concluded that the Recall, Precision, and F-measure values of the anchor-free quadrilateral detector are higher than those of the anchor-based quadrilateral detector. To further illustrate the performance differences, the detection results of the five quadrilateral detectors on the Instrument Dataset test set are visualized in Figure 12. It can be observed that TextBoxes++ and S2A-Net cannot accurately detect the third instrument image reading. The TextBoxes++ algorithm has a cross-connection situation, which is clearly caused by a problem with the sequential protocol. The other two detectors have issues with incomplete readings and excessive background information. On the instrument images with perspective transformation, the proposed method can still accurately detect the instrument readings with almost no background information, and the detection boxes are not cross-connected. These results verify that the proposed method effectively addresses the challenges posed by perspective transformation in instrument images while avoiding the complications associated with complex sequential protocols.

## 5. Conclusions

In this paper, a novel QCDNet method is proposed for detecting industrial instrument readings based on MsFPN and PCDR. In contrast to the existing detectors, QCDNet detects the contours of instrument readings in a disentangled manner, which exploits geometric properties to improve detection performance. Firstly, QCDNet utilizes MsFPN to fuse the extracted multi-scale features to obtain strong semantic feature information. Subsequently, PCDR is utilized to disentangle the parameters of the linear equations for each side of the instrument read contour in polar coordinates. By enhancing the geometric properties of instrument contour sides through customized Polar IoU loss and cosine angle loss, QCDNet can independently learn the representation of each disentangled side. Finally, extensive experiments were carried out on the Instrument Dataset, and the qualitative and quantitative results show the effectiveness of QCDNet for instrument read detection. The comparison results with the existing detectors verify that QCDNet can overcome the effects caused by rotation and perspective transformation. Meanwhile, compared with existing quadrilateral detectors, QCDNet solves the problem of vertex entanglement without relying on complex sequential protocols. Therefore, it can be concluded that QCDNet has the potential to be applied to detect instrument readings in real industrial scenarios. Considering the complexity and diversity of industrial instrument, in future research, we will improve the detection box of the proposed method so that the detection object can be extended to all kinds of instruments, including pointer meters with different shapes, and continuously enhance the detection performance of our method.

## Figures and Tables

**Table 1:** The results of the ablation experiment.

MsFPN	Polar-IoU Loss	Cosine Angle Loss	F-Measure
			0.8741
√			0.9045
	√		0.9285
		√	0.9215
√	√	√	0.9489

**Table 2:** The results of the ablation experiment for MSFPN.

Method	AP50	AP85
Resnet50 with FPN	1	0.8739
Resnet50 with MsFPN	1	0.8858

**Table 3:** AP values for 5 methods at different IoU thresholds and inference time at IoU = 0.85.

Method	AP(0.5:0.95)	AP50	AP85	Inference Time (ms)
SSD	0.8452	1	0.8732	15.63
YOLO	0.8461	1	0.8813	25.87
RetinaNet	0.8483	1	0.8895	45.37
Faster RCNN	0.8474	1	0.8846	48.66
Proposed	0.8494	1	0.8858	46.81

**Table 4:** Performance comparison of six methods under different IoU thresholds.

Method	IoU = 0.5			IoU = 0.85		
	Recall	Precision	F-Measure	Recall	Precision	F-Measure
Rotated RetinaNet	0.9613	0.9674	0.9643	0.8582	0.9368	0.8958
Rotated Faster-RCNN	0.9679	0.9866	0.9772	0.8953	0.9531	0.9233
Gliding Vertex [18]	0.9703	0.9778	0.9740	0.8837	0.9484	0.9149
Oriented R-CNN [19]	0.9864	0.9889	0.9876	0.9164	0.9647	0.9399
RoI Transformer [20]	0.9736	0.9821	0.9778	0.9013	0.9358	0.9182
Proposed	0.9752	0.9983	0.9867	0.9255	0.9735	0.9489

**Table 5:** Performance comparison between the proposed method and four quadrilateral detection methods.

Method	Anchor Style	Recall	Precision	F-Measure
TextBoxes++ [26]	anchor-based	0.7483	0.8664	0.8030
SASM_Reppoints [31]	anchor-free	0.9075	0.9328	0.9200
S2A-Net [34]	anchor-based	0.8857	0.9164	0.9008
SASM_S2ANet [35]	anchor-based	0.8970	0.9265	0.9115
Proposed	anchor-free	0.9255	0.9735	0.9489

## Data Availability

The data presented in this study are available on request from the corresponding author.
